# De-Novo Stress Urinary Incontinence After Apical Prolapse Surgery: Potential Link with the Zone of Critical Elasticity

**DOI:** 10.3390/jcm14228153

**Published:** 2025-11-17

**Authors:** Yaman Degirmenci, Ceren Efe Sayın, Ina Shehaj, Mona Wanda Schmidt, Gilbert Georg Klamminger

**Affiliations:** 1 Department of Obstetrics and Gynecology, University Medical Center of the Johannes Gutenberg University Mainz, 55131 Mainz, Germany; mona.schmidt@unimedizin-mainz.de (M.W.S.); gilbert.klamminger@unimedizin-mainz.de (G.G.K.); 2 Department of Biostatistics and Medical Informatics, Faculty of Medicine, Kahramanmaraş Sütçü İmam University, 46040 Kahramanmaraş, Türkiye; cerenefe.stat@gmail.com; 3 Department of Obstetrics and Gynecology, University of Frankfurt, 60590 Frankfurt am Main, Germany; inashehaj@hotmail.com

**Keywords:** pelvic organ prolapse, stress urinary incontinence, de-novo stress urinary incontinence, integral theory, zone of critical elasticity

## Abstract

**Background/Objectives**: Pelvic organ prolapse (POP) surgery can lead to postoperative stress urinary incontinence (SUI) in previously continent women, termed de novo SUI. This study assessed the incidence and risk factors of de novo SUI after apical POP repair, hypothesizing that reduced bladder neck elasticity—particularly within the zone of critical elasticity (ZCE) described by the Integral Theory—contributes to its development. **Methods**: A retrospective single-center analysis was performed in 206 postmenopausal women (≥60 years) who underwent apical POP surgery without concomitant anti-incontinence procedures. Patients were classified by surgical approach as laparoscopic sacrocolpopexy (SCP) or vaginal native tissue repair. **Results**: The overall incidence of de novo SUI was 8.7%. Laparoscopic SCP for vaginal vault prolapse was significantly associated with a higher risk of postoperative SUI (OR 10.37, 95% CI 2.70–39.79, *p* = 0.001), whereas other procedures showed no significant association. Neither prior hysterectomy nor cystocele stage was an independent predictor of de novo SUI. **Conclusions**: These results suggest that surgical alteration of the ZCE—particularly excessive tension or reduced elasticity near the bladder neck—may impair urethral closure. Therefore, preserving ZCE integrity and carefully adjusting mesh tension during apical POP repair may reduce the risk of de novo SUI.

## 1. Introduction

Pelvic floor disorders, including urinary and fecal incontinence and pelvic organ prolapse (POP), affect up to half of all women, with increasing prevalence with age [1]. POP is particularly common in parous women, and about 5–10% report symptomatic awareness [2,3,4]. Up to half of women (49%) with POP have coexisting stress urinary incontinence (SUI) [4]. However, symptom frequency tends to decrease with advancing prolapse stage, likely due to urethral kinking [4,5,6], suggesting that, in some cases, prolapse may even appear to ‘protect’ from SUI symptoms.

When stress urinary incontinence becomes apparent only after reduction of the prolapse in otherwise continent women, it is referred to as ‘occult SUI’ (OSUI), thought to result from the correction of anatomic urethral kinking or prolapse-related obstruction. Women with OSUI have a higher risk of developing postoperative SUI [7]. Reported OSUI prevalence varies widely (6–83%) depending on the population and diagnostic methods, and therefore POP surgery may lead to postoperative SUI in up to half of cases [5,8,9,10].

The CARE and OPUS randomized controlled trials, which specifically addressed this question, showed that prophylactic anti-incontinence procedures performed during vaginal or abdominal prolapse repair can effectively decrease the risk of postoperative SUI. Nevertheless, nearly one-quarter of women still developed stress incontinence despite having an additional colposuspension or midurethral sling (MUS) in these study cohorts [11,12]. In contrast, one-third of women with pre-existing SUI experience resolution after POP surgery alone [13]. These findings highlight the complex interplay between POP and SUI, as well as the challenges of managing concomitant continence procedures. A 2018 Cochrane review found no conclusive difference in postoperative SUI rates between vaginal procedures with or without concomitant continence surgery, and data for sacrocolpopexy with versus without Burch colposuspension remains insufficient [14]. Building on CARE and OPUS, predictive models such as the SUI Risk Calculator were developed to estimate the risk of postoperative SUI based on patient characteristics [10]. However, studies show limited predictive accuracy across different cohorts and procedures [15,16,17].

Of note, preoperatively continent women with POP and no symptomatic or occult SUI may even develop postoperative SUI. Despite the lack of a standardized definition, this condition is commonly termed ‘de novo stress urinary incontinence’ [14], reported in about 10–25% of women undergoing POP surgery [18,19]. While postoperative SUI in women with OSUI is thought to result from correction of urethral kinking or prolapse-related obstruction, the mechanisms underlying de novo SUI in women without OSUI remain poorly understood. Furthermore, inconsistencies in preoperative diagnostic protocols make it difficult to distinguish OSUI-related postoperative SUI from ‘de novo SUI’, i.e., a loss of continence induced by treatment.

The pathophysiology of SUI is complex and multifactorial, involving bladder neck and urethral incompetence, impaired support, and levator ani dysfunction [20]. Over the past five decades, these mechanisms have been the subject of extensive research, forming the basis for the evolution of surgical concepts in the management of SUI. Earlier theories, such as the pressure transmission and hammock theories [21,22], while informative, proved insufficient to fully explain urinary continence. Ulmsten and Petros subsequently advanced the Integral Theory, later refined into the Integral System, which frames the pelvic floor as an integrated connective-tissue structure governed by vector forces [23]. It emphasizes that restoring anatomy restores function and describes the vagina as having dual roles: transmitting muscle forces for bladder neck and urethral closure and preventing urgency by supporting hypothesized stretch receptors in the proximal urethra and trigone. Anatomically, the bladder, urethra, vagina, uterus, bowel, and endopelvic fascia are regarded as a functional unit that must be considered as such. This theory underscores the importance of coordinated pelvic support in maintaining continence and provides a conceptual framework linking anatomic restoration to functional outcomes.

The Integral System proposes that bladder neck opening during voiding results from coordinated vector forces, with posterior compartment muscles actively opening the proximal urethra, underscoring the importance of balanced muscular interactions [24]. Within this framework, the authors defined the ‘zone of critical elasticity’ (ZCE), mainly corresponding to the Pelvic Organ Prolapse Quantification (POP-Q) point Aa, as crucial for effective muscle-driven voiding and urinary continence. Loss of elasticity in this area—whether from surgery or fibrosis—shifts the balance toward stronger posterior forces from the levator plate and longitudinal muscle of the anus (LMA), leading to refractory incontinence, termed ‘tethered vagina syndrome’ (TVS). Supporting this concept, studies have shown that restoring the ZCE with suburethral grafts can re-establish elasticity and cure incontinence resulting from loss of elasticity in the ZCE [25].

Although the precise anatomical mechanisms remain incompletely understood, accumulating evidence indicates that de novo SUI is closely linked to overcorrection of the bladder neck or POP-Q point Aa [26]. Kato et al. described the so-called “*central road*”—a cystoscopic finding observed after sacrocolpopexy in a patient with severe postoperative SUI—where a mesh strand at the trigone or posterior bladder base corresponded to the ZCE [27]. This observation suggested that disruption of the ZCE may impair the urethral closure mechanism and predispose to de novo SUI. A subsequent case-based study highlighted the role of mesh tension, reporting that intraoperative adjustment to avoid the ‘central road’ and bladder neck opening prevented de novo SUI after laparoscopic sacrocolpopexy. Notably, none of the women without baseline SUI developed postoperative SUI when such adjustment was performed, again implicating the ZCE, or POP-Q point Aa, as a key factor [28].

The lifetime risk for women undergoing surgical intervention for POP or incontinence is considerable, at approximately 11%, with reoperation rates of about 30% [29,30]. As POP and SUI surgeries are performed to improve quality of life rather than to treat life-threatening conditions, postoperative complications such as de novo SUI represent a significant clinical and emotional burden for patients and a persistent challenge for surgeons. In this study, we aimed to evaluate de novo SUI after apical POP repair and to explore its association with the bladder neck from the perspective of Integral Theory and the ZCE. Specifically, we investigated the hypothesis that bladder neck hypoelasticity, resulting from surgical scarring, particularly in the ZCE, plays a central role in the pathogenesis of de novo SUI after apical POP surgery.

## 2. Materials and Methods

This retrospective, single-center study analyzed data from postmenopausal women who underwent surgery for apical pelvic organ prolapse without concomitant anti-incontinence procedures. Two primary groups were defined according to surgical approach: laparoscopic and vaginal.

**Figure 1:**
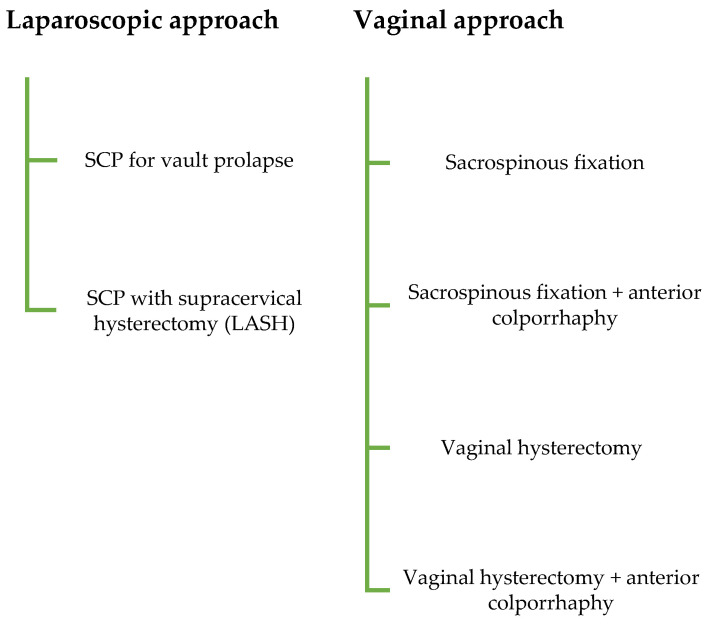
Flow chart illustrating patient selection and inclusion process for the study cohort.

All patients who underwent laparoscopic sacrocolpopexy for apical POP between 2012 and 2022 were identified from institutional records. To enable comparison of de novo SUI across surgical approaches, particularly the vaginal route, women who underwent vaginal native tissue repair for apical POP between 2018 and 2022 were also included. All relevant cases were identified through hospital surgical databases, and eligibility was verified by chart review. Standardized forms were used to collect data on preoperative and postoperative outcomes for eligible patients. Cases treated via the vaginal route with mesh were excluded to avoid confounding effects and due to the limited number of such procedures. Patients undergoing surgery for oncologic indications or suspected malignancy were likewise excluded. To reduce the potential influence of age on postoperative outcomes, only women aged 60 years or older were included. The laparoscopic group was further divided into two subgroups based on whether concomitant hysterectomy was performed. The vaginal group was stratified into four subgroups depending on the type of apical repair—vaginal hysterectomy or sacrospinous fixation—and the performance of a concomitant anterior colporrhaphy for cystocele (Flow-Chart, Figure 1).

Perioperative and clinical data were obtained from internal medical records. Baseline demographic and clinical variables included age, parity, menopausal status, preoperative POP stage, respectively, the stage of the cystocele according to POP-Q, body mass index (BMI), previous abdominal surgeries, previous hysterectomy, urogynecologic surgery, and the American Society of Anesthesiologists (ASA) classification. Additional data included symptoms of overactive bladder, length of hospital stay, and preoperative assessment of stress urinary incontinence (SUI). The latter was obtained through routine preoperative urogynecologic evaluation, which included a cough stress test and a structured assessment of SUI symptoms based on established clinical definitions.

In the laparoscopic group, sacrocolpopexy was performed either together with supracervical hysterectomy, or for post-hysterectomy vaginal vault prolapse, or as a subsequent step following concomitant total hysterectomy in one case. In the former, mesh fixation was predominantly performed at the cervical or apical level following supracervical hysterectomy. For vault prolapse, the mesh was primarily attached to the vaginal apex and predominantly in the anterior compartment. For the vaginal approach, standard vaginal cuff fixation to the uterosacral ligaments was performed following hysterectomy, typically in the context of McCall culdoplasty when sacrospinous fixation was not selected for apical support. In case of cystocele, concurrent anterior colporrhaphy was performed. In patients with post-hysterectomy vaginal vault prolapse, sacrospinous fixation was consistently carried out.

All procedures were carried out by urogynecologic surgeons or under their direct supervision. Postoperative follow-up data were available for all included cases and included urogynecologic examinations and assessments of stress urinary incontinence (SUI).

The primary objective of the study was to determine the incidence of de novo SUI following apical POP surgery and to compare outcomes between laparoscopic and vaginal approaches, with additional subgroup analyses conducted to test the study hypothesis further.

De novo SUI was defined according to the prevailing literature as the occurrence of stress urinary incontinence in women who were continent prior to surgery, based on preoperative clinical evaluation (history and physical examination), but who subsequently developed SUI symptoms postoperatively. Statistical analyses were performed using SPSS version 20. The Shapiro–Wilk test was used to assess the normality of continuous variables. Non-normally distributed continuous variables were analyzed using the Mann–Whitney U test and presented as medians with ranges. Categorical variables were compared using Pearson’s chi-square test or Fisher’s exact test, as appropriate, and reported as *n* (%). Statistical significance was set at *p* < 0.05. Variables with *p* < 0.05 in the univariate analysis were subsequently entered into a multiple logistic regression model. Odds ratios (ORs) with 95% confidence intervals (CIs) were calculated. The study was conducted in accordance with the principles of the Declaration of Helsinki (1964). According to local regulations for retrospective analyses of anonymized routine clinical data, such analyses do not require Ethics Committee approval. Therefore, no separate Ethics Committee approval was needed for this study.

## 3. Results

A total of 206 patients were included in the analysis. Among them, 58 underwent laparoscopic procedures: 18 patients received sacrocolpopexy combined with laparoscopic supracervical hysterectomy (LASH), and 40 patients underwent primary sacrocolpopexy in terms of vaginal vault fixation. A total of 148 patients treated via a vaginal approach were analyzed. Of these, the majority (*n* = 85) underwent vaginal hysterectomy with concomitant anterior colporrhaphy. Only six patients underwent vaginal hysterectomy without bladder involvement or anterior colporrhaphy. In cases of apical POP, 35 patients received apical fixation with sacrospinous ligament fixation along with anterior colporrhaphy, while 22 patients underwent sacrospinous ligament fixation without anterior colporrhaphy.

The demographic characteristics, along with descriptive statistics, are presented in Table 1. Comparisons of variables and their effects on the occurrence of de novo stress urinary incontinence (SUI) are shown in Table 2. The median age of the patients was 71.2 years (range, 60.2–88.7), with a median parity of 2 (range, 0–6). Approximately one-third (30.5%) were presented with advanced POP-Q stage III or IV. Cystocele was most frequently classified as stage II (58.6%), followed by stage III or higher (31.0%) and stage I (10.3%). Preoperatively, overactive bladder (OAB) symptoms were reported by 55.1% of women, the majority of whom exhibited OAB-dry (76.1%) rather than OAB-wet (23.9%). The median BMI was 26.2 kg/m^2^ (range, 17.4–42.0). A total of 36.4% of patients had undergone a prior hysterectomy, and 25.7% had prior POP surgery.

Variables found significant in univariate analyses (Table 2) were subsequently included in a multiple logistic regression model. These included prior hysterectomy, prior POP surgery, and the surgical group. The overall model was statistically significant (*p* = 0.002) with moderate explanatory power (Nagelkerke R^2^ = 0.236).

A prior hysterectomy was not an independent predictor of de novo SUI following apical POP surgery (*p* = 0.251; OR = 5.076, 95% CI: 0.317–83.333). Although the odds ratio indicated a fivefold increased risk, the wide confidence interval and lack of significance warrant cautious interpretation. Similarly, prior POP surgery was not identified as an independent risk factor for de novo SUI (*p* = 0.557; OR = 0.465, 95% CI: 0.064–3.387). The surgical group as a whole was not statistically significant (*p* = 0.342). However, sacrocolpopexy for vaginal vault prolapse showed a substantial association with de novo SUI (OR = 26.152, 95% CI: 1.690–404.584, *p* = 0.020). These regression results are not presented in tabular form.

When analyzing the impact of surgical technique on de novo SUI (Table 3), with vaginal hysterectomy with anterior colporrhaphy set as the reference, the logistic regression model was statistically significant (*p* = 0.016), indicating a relevant influence of surgical type. Laparoscopic sacrocolpopexy for vault prolapse was significantly associated with increased odds of de novo SUI compared to vaginal hysterectomy with anterior colporrhaphy (OR = 10.368, 95% CI: 2.701–39.794, *p* = 0.001). This suggests that sacrocolpopexy in the setting of vault prolapse may represent a risk factor for postoperative SUI and emphasizes the importance of surgical technique in prolapse management. Sacrospinous ligament fixation with anterior colporrhaphy and sacrocolpopexy with concomitant laparoscopic-assisted supracervical hysterectomy (LASH) demonstrated non-significant trends toward increased risk (OR = 2.562, 95% CI: 0.491–13.365 and OR = 1.608, 95% CI: 0.158–16.405, respectively). Sacrospinous fixation without anterior colporrhaphy and vaginal hysterectomy without anterior colporrhaphy both yielded extremely low, non-significant odds ratios (OR ≈ 0), likely due to sparse data, rendering estimates unreliable.

The impact of preoperative cystocele stage on de novo SUI was also analyzed using logistic regression (Table 3), with Stage 1 cystocele as the reference. Although patients with Stage 2 and Stage 3+ cystocele demonstrated increased odds compared to Stage 1, none of these associations reached statistical significance. Stage 2 cystocele showed an OR of 1.45 (95% CI: 0.170–12.323, *p* = 0.735), while Stage 3+ cystocele showed an OR of 2.13 (95% CI: 0.238–18.951, *p* = 0.500). The overall effect of preoperative cystocele stage on de novo SUI was not statistically significant (*p* = 0.699).

## 4. Discussion

De novo stress urinary incontinence (SUI) following apical POP surgery is hypothesized to be associated with loss of bladder neck elasticity—particularly within the zone of critical elasticity (ZCE) described by the Integral Theory—which plays a central role in maintaining urethral closure and continence. Although de novo SUI may occur after any POP repair, postoperative urethral unkinking alone does not fully explain this phenomenon, suggesting that additional mechanisms are likely involved.

Studies assessing outlet obstruction using the Blaivas nomogram found that women who developed de novo SUI after transvaginal mesh surgery had only slightly higher preoperative obstruction rates, with most cases (≈80%) showing mild or no obstruction. These findings further suggest that significant outlet obstruction is not required for de novo incontinence and imply that mechanisms other than urethral kinking or obstruction contribute to postoperative SUI [31].

To further explore whether de novo incontinence results from disruption of bladder neck function, the predictive value of prolapse reduction tests provides valuable insight. In women with occult SUI, reduction of the prolapse may unmask leakage due to temporary urethral unkinking. The fact that the positive predictive value (PPV) of such prolapse reduction tests is below 100% suggests that continence may be restored following anatomical correction, consistent with the Integral Theory. Conversely, a high but not absolute negative predictive value (NPV) indicates that most women who remain continent during testing will stay continent postoperatively, while a small proportion may develop de novo SUI due to newly acquired dysfunction—potentially from altered bladder neck elasticity as hypothesized in the present study. Reported NPVs for native tissue repair (NTR) in the anterior compartment are approximately 93%, consistent with our findings [9]. In contrast, data from the CARE trial for sacrocolpopexy demonstrated PPV and NPV values of approximately 60% in the non-Burch group and an NPV of nearly 80% in the Burch group, which are likewise concordant with our results and align with this theoretical framework [32]. Differences in the sensitivity and predictive value of various prolapse reduction tests—such as those using a pessary, swab, forceps, or speculum—may reflect their differing effects on bladder neck and anterior vaginal wall dynamics [32]. Reduced elasticity of the ZCE can be demonstrated by a provocative test in which proximal displacement of the apical anterior vaginal wall induces stress incontinence. A similar effect occurs during speculum insertion, which, unlike pessary testing, not only corrects the prolapse but also alters ZCE elasticity. This mechanism may explain the higher predictive value of speculum-based compared with pessary-based reduction tests [25].

Ultrasound studies demonstrate that POP surgery can straighten the urethra and reduce its postoperative mobility compared with preoperative status. This morphological change has been linked to de novo SUI, suggesting that loss of physiological urethral curvature and reduced ZCE elasticity may impair urethral closure and contribute to postoperative dysfunction [33].

The overall incidence of de novo SUI in our cohort (8.7%) was consistent with previous reports across different POP procedures. Sacrocolpopexy for vaginal vault prolapse was the only operation significantly associated with increased postoperative SUI risk (OR > 10.3), consistent with prior studies reporting rates of 17–24% after sacral suspension procedures [19,34,35]. The higher risk appears to result from urethrovesical straightening and loss of bladder neck elasticity rather than prolapse stage.

In our cohort, a prior hysterectomy was significant in univariate analysis but did not remain an independent predictor of de novo SUI following apical POP surgery (*p* = 0.251; OR = 5.076). Nevertheless, this observation supports the underlying hypothesis. According to the refined Integral Theory by Ulmsten and Petros, the pelvic floor functions as an integrated connective tissue network regulated by vector forces, with the region surrounding the bladder neck representing a critical zone in which tissue elasticity is essential for maintaining normal continence and bladder emptying [23]. Although prior hysterectomy alone does not predispose to urinary incontinence [36], the Integral Theory suggests that preexisting laxity of pelvic support structures and surgical manipulation near the ZCE may impair physiological function. This risk may be further aggravated by loss of elasticity, particularly in cases involving mesh implantation.

The literature likewise indicates that prior hysterectomy does not affect de novo SUI risk after transvaginal mesh (TVM) procedures but may influence outcomes after sacral fixation. Thus, it appears that postoperative incontinence is not caused by hysterectomy itself but rather by subsequent anatomical and functional alterations in the anterior compartment and urethrovesical junction [37]. Supporting this concept, Yoshio et al. reported a de novo SUI rate of approximately 17% after laparoscopic sacrocolpopexy, with only 2% of their cohort having undergone hysterectomy. This lower rate may indicate that sacrocolpopexy after hysterectomy carries a higher risk, likely due to altered bladder neck elasticity and urethrovesical alignment [19]. Similarly, Kim et al. observed an 18% de novo SUI rate without distinguishing hysterectomy status. However, the proportion of women with a previous hysterectomy was higher in the de novo group, although not statistically significant [38].

Secondary analysis of the CARE data linked higher cystocele severity (POP-Q point Ba) with increased de novo SUI risk, whereas the OPUS trial found no such correlation, underscoring the complex interaction between anatomy and continence [39,40]. In our study, although women with Stage 2 or Stage 3+ cystocele had higher odds compared with Stage 1, none of these differences reached statistical significance (*p* = 0.699). Anatomical evidence suggests that, rather than point Ba, point Aa of the POP-Q system—corresponding to the bladder neck and the zone of critical elasticity (ZCE)—is more directly related to postoperative stress incontinence [10,37,41]. Notably, overcorrection at point Aa has been shown to increase the risk of postoperative SUI, and studies assessing the ΔAa value (pre- to postoperative change) have identified it as a key determinant of de novo SUI. These findings support the Integral Theory, emphasizing that overtension and reduced elasticity within the ZCE may disrupt the continence mechanism [26]. Thus, the type of surgery and the anatomical changes it induces appear to be the principal factors influencing postoperative continence. The omission of point Aa—the bladder neck reference point—in the CARE analysis may partly explain its contrasting results.

In the study by Kim et al., which examined de novo SUI after sacrocolpopexy, previous transvaginal mesh surgery was identified as an independent predictor (aOR 8.92; 95% CI 1.50–52.78), whereas the Ba point showed no association with its development. These findings highlight the importance of surgical history over anatomical staging in predicting postoperative continence outcomes [38]. The authors suggested that overtensioning of the urethrovesical junction after sacrocolpopexy—particularly in women with prior mesh placement—may underlie this increased risk. Similarly, other reports, although showing no significant postoperative change in point Aa, propose that mesh shrinkage can exert excessive tension on the bladder neck, contributing to de novo SUI following mesh surgery [18].

In light of these observations, the Integral Theory remains highly relevant in explaining the pathophysiology of de novo SUI after POP surgery, underscoring the importance of preserving elasticity and functional integrity within the ZCE as defined in the tethered vagina syndrome framework. A recent case-based study further highlighted the significance of proper mesh tension in preventing de novo SUI [28]. Thus, meticulous intraoperative adjustment of mesh tension and preservation of ZCE elasticity may help reduce the risk of postoperative incontinence following apical POP repair.

Compared with laparoscopic or abdominal procedures, vaginal NTR is associated with lower rates of de novo SUI—reported at about 11% overall and only 4% among women requiring treatment [42,43]. Consistent with the “tethered vagina syndrome,” loss of elasticity within the ZCE—for example, after vaginal mesh surgery—appears to increase the risk of postoperative SUI relative to NTR. Meta-analytic data further confirm that anterior armed mesh repair elevates this risk [14]. A study examining the relationship between Point Aa, mesh placement, and tissue elasticity limited mesh fixation during sacrocolpopexy to the apical and posterior vaginal wall, avoiding anterior dissection. Although this approach led to higher cystocele recurrence rates, the incidence of de novo SUI remained low (7.5%), supporting the role of anterior wall elasticity in continence preservation [44]. Similarly, in a small cohort undergoing laparoscopic supracervical hysterectomy with sacrocolpopexy—where the ZCE was presumably preserved—the postoperative de novo SUI rate was 0%. Interestingly, concomitant Burch colposuspension did not prevent but instead worsened continence outcomes. These findings collectively emphasize the pivotal role of preserving the ZCE in preventing de novo SUI after apical POP surgery [45].

Our study demonstrated that isolated apical fixation with sacrospinous ligament fixation (SSLF) was not associated with de novo SUI. Although odds were slightly higher when the anterior vaginal wall was included, the difference was not statistically significant compared with vaginal hysterectomy with anterior colporrhaphy. The vector changes inherent to sacrospinous fixation may modestly alter the physiological force directions described in the Integral Theory, yet without a measurable impact on continence outcomes. Consistent with our findings, previous studies have shown that adding SSLF to vaginal prolapse repair does not increase de novo SUI incidence, suggesting that apical fixation alone is unlikely to compromise continence mechanisms [46]. Although age appears to influence the individual risk of developing de novo SUI, the effects of BMI and diabetes remain controversial [10,34]. In our study, age was not identified as a risk factor; however, to minimize potential bias, only patients aged 60 years and older were included in the analysis.

Despite its strengths, our study has limitations that should be acknowledged. Due to its retrospective design, it carries inherent risks of selection and information bias. Although all cases were classified using the POP-Q system, the lack of precise centimeter-based measurements precluded more detailed analysis. Urodynamic testing was not routinely performed, which could have provided additional physiological insight, and the absence of a validated symptom questionnaire may have affected the accuracy of subjective SUI reporting. Additionally, the relatively small sample size—especially in the sacrocolpopexy subgroup—limits external generalizability in these aspects. Therefore, a post hoc power calculation was performed using G*Power v3.1 for the primary comparison between surgical groups (*n* = 206; α = 0.05; two-tailed). Based on the observed difference in de novo SUI incidence (~24%), the calculated power was approximately 80%, indicating that the sample size was likely sufficient to detect clinically relevant differences between groups. Absence of statistical significance is more likely to reflect true clinical equivalence rather than insufficient statistical sensitivity.

## 5. Conclusions

In conclusion, our findings underscore the importance of preserving elasticity and functional integrity at the bladder neck and the zone of critical elasticity (ZCE) during apical POP repair. Tailoring surgical tension and respecting anatomic balance may help prevent postoperative de novo SUI, improving both functional and quality-of-life outcomes.

## Figures and Tables

**Table 1:** Baseline demographic and clinical characteristics of the study population.

Variable	Descriptive Statistics *
Age (years)	71.2 (60.2–88.7)
Parity	2 (0–6)
POP-Q Stage 3 or 4	62 (30.5)
Cystocele	
Stage I	18 (10.3)
Stage II	102 (58.6)
Stage III and over	54 (31)
OAB Symptoms preoperatively	
No	92 (44.9)
Yes	113 (55.1)
OAB-Wet	27 (23.9)
OAB-Dry	86 (76.1)
Previous abdominal surgery (*n*)	1 (0–7)
Previous hysterectomy	
Yes	75 (36.4)
No	131 (63.6)
Previous POP surgery	
Yes	53 (25.7)
No	153 (74.3)
Previous urinary incontinence surgery	
Yes	7 (3.4)
No	199 (96.6)
BMI (kg/m^2^)	26.2 (17.4–41.9)
Length of hospital stay (days)	5 (2–11)
ASA Classification	2 (1–3)
De novo SUI	
Yes	18 (8.7)
No	188 (91.3)
Follow-up postoperatively (Day)	67.8 (27–398)

* Continuous data are presented as median (range), and categorical data as *n* (%). BMI, body mass index; POP-Q, Pelvic Organ Prolapse Quantification system; OAB, overactive bladder; ASA, American Society of Anesthesiologists classification; SUI, Stress urinary incontinence.

**Table 2:** Comparison of demographic and clinical variables between patients with and without de novo stress urinary incontinence.

Variable	Descriptive Statistics		*p* *
	SUI	No SUI	
Age (years)	68.0 (61.4–81.6)	71.3 (60.2–88.7)	0.350
Parity	2 (1–3)	2 (0–6)	0.330
POP-Q Stage 3 or 4			0.430
Yes	7 (11.3)	55 (88.7)	
No	11 (7.8)	130 (92.2)	
Cystocele			0.402
Stage I	1 (5.6)	17 (94.4)	
Stage II	8 (7.8)	94 (92.2)	
Stage III and over	6 (11.1)	48 (88.9)	
OAB Symptoms preoperatively			0.954
Wet	2 (7.4)	25 (92.6)	
Dry	8 (7.6)	78 (90.7)	
None	8 (8.7)	84 (83.9)	
Previous abdominal surgery (*n*)	0.50 (0–3)	1(0–7)	0.370
BMI (kg/m^2^)	26.81 (22.0–31.8)	25.97 (17.4–41.9)	0.287
ASA Classification	2 (2–3)	2 (1–3)	0.968
Previous hysterectomy			0.037
Yes	11 (14.7)	64 (85.3)	
No	7 (5.3)	124 (94.7)	
Previous POP surgery			0.008
Yes	10 (18.9)	43 (81.1)	
No	8 (13.4)	145 (94.8)	
Previous urinary incontinence surgery			0.478
Yes	1 (14.3)	6 (85.7)	
No	17 (8.5)	182 (91.5)	
Length of hospital stay (days)	5 (3–8)	5 (2–11)	0.756
Follow-up postoperatively (Day)	61 (36–192)	69 (27–398)	0.068
Study Group			<0.001
Sacrospinous fixation	0 (0)	22 (100)	
Sacrospinous fixation + anterior colporrhaphy	3 (8.6)	32 (91.4)	
SCP with supracervical hysterectomy (LASH)	1 (5.6)	17 (94.4)	
SCP for vault prolapse	11 (27.5)	29 (72.5)	
Vaginal hysterectomy	0 (0)	6 (100)	
Vaginal hysterectomy + anterior colporrhaphy	3 (3.5)	82 (96.5)	

* Continuous data are presented as median (range); categorical data as *n* (%). Statistical comparisons were performed using the Mann–Whitney U or Chi-square test, as appropriate. BMI, body mass index; POP-Q, Pelvic Organ Prolapse Quantification system; OAB, overactive bladder; ASA, American Society of Anesthesiologists classification; SUI, Stress urinary incontinence; SCP, Sacrocolpopexy; LASH, laparoscopic-assisted supracervical hysterectomy.

**Table 3:** Multivariate logistic regression analysis of independent risk factors for de novo stress urinary incontinence.

Risk Factor (Ref. Category)	OR	%95 Confidence Interval	*p* *
Surgical technique			0.016
Vaginal hysterectomy + anterior colporrhaphy (ref)	1.00	-	-
SCP for vault prolapse	10.368	2.70–39.79	0.001
SCP with supracervical hysterectomy (LASH)	1.608	0.15–16.40	0.68
Sacrospinous fixation + anterior colporrhaphy	2.562	0.49–13.36	0.26
Sacrospinous fixation	~0	-	-
Vaginal hysterectomy	~0	-	-
Cystocele stage			0.699
Stage I (ref)	1.00	-	-
Stage II	1.45	0.17–12.32	0.735
Stage III and over	2.13	0.23–18.95	0.500

* Multivariate logistic regression including variables significant in univariate analysis. Reference categories are indicated in parentheses. Statistical significance was set at *p* < 0.05.

## Data Availability

The original contributions presented in this study are included in the article. Further inquiries can be directed to the corresponding author.
